# Acute ethanol treatment upregulates *th1, th2*, and *hdc* in larval zebrafish in stable networks

**DOI:** 10.3389/fncir.2013.00102

**Published:** 2013-05-31

**Authors:** Henri A. J. Puttonen, Maria Sundvik, Stanislav Rozov, Yu-Chia Chen, Pertti Panula

**Affiliations:** Neuroscience Center and Institute of Biomedicine/Anatomy, University of HelsinkiHelsinki, Finland

**Keywords:** zebrafish, ethanol, tyrosine hydroxylase, histidine decarboxylase, behavior

## Abstract

Earlier studies in zebrafish have revealed that acutely given ethanol has a stimulatory effect on locomotion in fish larvae but the mechanism of this effect has not been revealed. We studied the effects of ethanol concentrations between 0.75 and 3.00% on 7-day-old larval zebrafish (*Danio rerio*) of the Turku strain. At 0.75-3% concentrations ethanol increased swimming speed during the first minute. At 3% the swimming speed decreased rapidly after the first minute, whereas at 0.75 and 1.5% a prolonged increase in swimming speed was seen. At the highest ethanol concentration dopamine levels decreased significantly after a 10-min treatment. We found that ethanol upregulates key genes involved in the biosynthesis of histamine (*hdc*) and dopamine (*th1* and *th2*) following a short 10-min ethanol treatment, measured by qPCR. Using *in situ* hybridization and immunohistochemistry, we further discovered that the morphology of the histaminergic and dopaminergic neurons and networks in the larval zebrafish brain was unaffected by both the 10-min and a longer 30-min treatment. The results suggest that acute ethanol rapidly decreases dopamine levels, and activates both forms or *th* to replenish the dopamine stores within 30 min. The dynamic changes in histaminergic and dopaminergic system enzymes occurred in the same cells which normally express the transcripts. As both dopamine and histamine are known to be involved in the behavioral effects of ethanol and locomotor stimulation, these results suggest that rapid adaptations of these networks are associated with altered locomotor activity.

## Introduction

Ethanol is the most widely used recreational drug. Ethanol abuse has been associated with many diseases of the brain and hazardous behavior, and due to its increasing prevalence, it is crucial to understand the effects of ethanol on the brain in detail. The dopaminergic system is one of the many targets of ethanol, and it has been widely studied in several models. Earlier studies have shown that the dopaminergic neurons in the VTA in rats are activated by ethanol administration (Gessa et al., 1985). Furthermore, studies have shown that the extracellular dopamine concentration in target areas of these neurons, mainly the nucleus accumbens, increases after ethanol exposure both in rats (Di Chiara and Imperato, 1986; Yoshimoto et al., 1992) and in mice (Tang et al., 2003). The activation of this mesolimbic dopamine pathway has been linked to mechanisms of substance addiction and reinforcement (Di Chiara and Imperato, 1988) and the increase in locomotor activity observed upon administration of ethanol in rodents (Cohen et al., 1997). Although the changes in dopamine levels are well known, the acute effect of ethanol on the expression of tyrosine hydroxylase (TH), the rate-limiting enzyme in dopamine synthesis, is not well-understood, with some evidence reporting an increase in *th* mRNA levels (Oliva et al., 2008). A chronic treatment with ethanol has been shown to increase *th* mRNA levels in the rat brain (Lee et al., 2005; Navarrete et al., 2013), but there is also evidence suggesting that TH protein levels decrease after chronic ethanol treatment (Kashem et al., 2012).

In mammals, the forebrain nucleus accumbens has been considered a critical region for transforming motivational and cognitive inputs to action since the initial concept was presented (Mogenson et al., 1980). Dopamine release in this region is needed for the locomotor activation induced by stimulatory drugs in mammals (Kelly et al., 1975; Farrar et al., 2010). The related functional neural circuits have not been analyzed in zebrafish, although both low concentrations of alcohol and d-amphetamine in swimming water significantly increase locomotor activity in larval zebrafish (Irons et al., 2010). Since the zebrafish do not possess dopaminergic neurons in the mesencephalon, significant efforts have been devoted to identifying the origin of subpallial dopaminergic inputs which might correspond to the mammalian striatonigral dopaminergic system necessary for initiation of voluntary movements. Both in adult zebrafish (Kaslin and Panula, 2001) and early larval zebrafish (McLean and Fetcho, 2004), local TH immunoreactive cells give rise to local projections. The TH-immunoreactive neurons in the posterior tuberculum also give rise to ascending projections toward the subpallium (Kaslin and Panula, 2001; Rink and Wullimann, 2001). A detailed analysis of individual neurons of all dopaminergic clusters using th:rasEGFP transgenic fish suggests that at an early stage (4 dpf) only very few dopaminergic neurons in the posterior tuberculum send ascending projections, whereas most of the fibers are of intrinsic subpallial origin (Tay et al., 2011). The current concept is that both intrinsic and ascending projections contribute to the dopaminergic inputs to subpallium. Many diencephalic dopaminergic cell clusters give rise to extensive descending projections to the rhombencephalon and spinal cord in both larval and adult zebrafish (Kaslin and Panula, 2001; McLean and Fetcho, 2004; Tay et al., 2011). Thus, dopamine can potentially regulate movement on all levels from telencephalon to spinal cord, where dopaminergic fibers are concentrated in the ventral horn (McLean and Fetcho, 2004).

Although less studied, the histaminergic system is known to be affected by ethanol. Acutely administered ethanol increases histamine content in rodents (Subramanian et al., 1978; Rawat, 1980), with one study reporting a decrease with higher ethanol doses (Papanicolaou and Fennessy, 1980). Histamine has also been associated with ethanol tolerance in rats, with ethanol sensitive alcohol non-tolerant rats showing lower levels of brain histamine than ethanol insensitive alcohol tolerant rats and decreased ethanol tolerance in alcohol tolerant rats upon pharmacological inhibition of histamine synthesis (Lintunen et al., 2002). Recent studies have also shown that knocking out the histamine synthesizing enzyme histidine decarboxylase (HDC) in mice inhibits the increase of locomotor activity caused by ethanol (Nuutinen et al., 2010), and mice treated with histamine H3 receptor antagonists or those lacking H3 receptor do not show ethanol-induced conditioned place preference (Nuutinen et al., 2011). In the light of these new discoveries, the histaminergic system can be considered a major area of interest in ethanol research.

A vast majority of reported ethanol studies have utilized different rodent models. Lately, the zebrafish has been established as an alternative model to study the effects of ethanol. Behavioral analysis methods suitable for ethanol research have been established both for adult (Gerlai et al., 2000; Kily et al., 2008; Mathur et al., 2011) and larval zebrafish (Lockwood et al., 2004). There are also several advantages to using zebrafish, including the possibility to visualize whole neurotransmitter networks *in vivo* in larval stages of the fish, as has been shown in several earlier studies (Chen et al., 2009; Sallinen et al., 2009b). An acute exposure of adult zebrafish to ethanol increases brain dopamine levels in a dose-dependent manner (Chatterjee and Gerlai, 2009), but the kinetics of these changes which potentially modify the functions of important circuits are not known. Furthermore, the regulation of the rate-limiting dopamine synthesizing enzyme TH has not been studied. We hypothesized that the increased dopamine levels should be associated with essential changes in synthesizing enzymes TH, and aimed at identifying which of the two TH forms is activated. We also hypothesized that synthesis of histamine, another amine involved in increased vigilance and motor activity, could undergo essential changes following ethanol exposure. In this study, we used 7-day-old larval zebrafish to study the effect of several acute short-term ethanol treatments on swimming behavior. In addition, we associated the changes observed to possible functional or anatomical alterations in key markers of the histaminergic and dopaminergic circuits using confocal analysis on whole neuronal networks and *in situ* hybridization.

## Materials and methods

### Experimental animals

Larval zebrafish of a wild-type Turku strain were used in all experiments. This strain has been maintained in the laboratory for more than a decade, and has been used in several earlier publications (Chen et al., 2009; Sallinen et al., 2009a). Fish were bred and maintained according to Westerfield (Westerfield, 2000). A permit for the experiments was obtained from the Office of the Regional Government of Southern Finland. All batches of embryos were obtained from several parent fish in order to minimize the chance of a rare genotype affecting the results.

### Ethanol treatment for histologic and quantitative methods

Ten to twenty 7dpf (days post-fertilization) larval zebrafish were transferred to a six-well plate in 1 × E3 (zebrafish embryonic medium; 5.00 mM NaCl, 0.44 mM CaCl_2_, 0.33 mM MgSO_4_ and 0.17 mM KCl). The 1 × E3 was then replaced with 1 × E3 containing ethanol. Four different ethanol concentrations were used: 0.00% (control), 0.75, 1.50, and 3.00% (v/v). These concentrations were chosen based on previous studies with zebrafish and ethanol (Lockwood et al., 2004; Chatterjee and Gerlai, 2009). The treatment durations used were 10 and 30 min. Following the ethanol treatment, the larvae were quickly collected into 1.5 ml microcentrifuge tubes and sacrificed on ice.

### Behavioral analysis

The locomotor activity of 7dpf larvae after ethanol exposure was observed during a 10-min time period. Forty eight larvae were tracked at a time on a 48-well plate, as described previously (Peitsaro et al., 2007). The larvae were divided into four treatment groups of 12 larvae. Ethanol solutions used were the same as those described above. The solution was administered carefully into the wells immediately prior to the tracking in order to assess the acute locomotor effect of ethanol exposure. When analysing total distance moved, fish that did not swim at all during the trial were considered outliers and excluded from the analysis. The amount of larvae excluded from each of the groups due to zero movement was as follows: control: 1 out of 48, 0.75%: 5 out of 61, 1.50%: 7 out of 68 and 3.00%: 2 out of 55. The values were transformed using log_10_ transformation, after which the values conformed better to a normal distribution and were analysed using 1-way ANOVA followed by Tukey's *post-hoc* test. Other variables were analysed without the use of any transformations using the same statistical tests. Additionally, total distance moved was also analysed with 1-min intervals, with statistical analysis done using Two-Way repeated measures (RM) ANOVA followed by Bonferroni's post hoc test after performing a log_10_ transformation as described above. Fish that did not move at all during one or more intervals were assigned the value 10^−4^ for total swimming distance during those time points to enable use of a log_10_ transformation.

### Quantitative PCR

Groups of 15 larvae were treated with ethanol for 10 min as described above. Total RNA was isolated using the RNeasy Mini Kit (Qiagen, Hilden, Germany), followed by cDNA synthesis using the SuperScriptIII kit (Invitrogen). The SmartCyclerII® cycling platform was used for qPCR. The reaction mix consisted of SYBR Green premix (Takara, Madison, WI, USA), primers and cDNA template. The primer sequences have been described earlier (Sallinen et al., 2010; Pavlidis et al., 2011). Quantification was done by Ct value comparison, using the Ct value of β-actin as an internal standard (Livak and Schmittgen, 2001). Statistical analysis was done using One-Way ANOVA followed by Tukey's *post-hoc* test.

### Tyrosine hydroxylase 1 and 2, and histidine decarboxylase whole-mount *in situ* hybridization

Antisense digoxigenin (DIG)-labeled RNA probes were synthesized using the DIG RNA labeling kit (Roche Diagnostics, Germany). The clones used have been described previously (Chen et al., 2009; Sundvik et al., 2011). Larvae samples for whole-mount *in situ* hybridization (WISH) were collected after ethanol treatment and fixed in 4% PFA (paraformaldehyde) in PBS overnight. *WISH* was carried out according to the Thisse lab protocol with minor modifications (Thisse and Thisse, 2008). The heads were dissected after fixation in order to expose the brain. All hybridization steps were done at 67°C. The probes were detected using NBT (nitro blue tetrazolium, Roche diagnostics GmBH, Mannheim, Germany)/BCIP (5-bromo 4-chloro 3-indolyl phosphate, Roche diagnostics GmBH, Mannheim, Germany).

### Tyrosine hydroxylase and histamine immunohistochemistry

Larvae were collected after ethanol treatment and fixed overnight in 4% EDAC (1-ethyl-3,3(dimethyl-aminopropyl) carbodiimide) and 0.1% PFA in PBS for tyrosine hydroxylase (TH1) and histamine double staining. Samples used only for TH1 staining were fixed in 4% PFA in PBS. The detailed protocol for immunohistochemistry has been described earlier (Sallinen et al., 2009b). For primary antibodies, monoclonal mouse anti-TH1 antibody (Diasorin/Immunostar, lot no. 22941) diluted 1:1000 and polyclonal rabbit anti-Histamine serum [rabbit anti-histamine 19C (Panula et al., 1990)] diluted 1:10000 were used. For detection, the samples were incubated with Alexa-conjugated antibodies (Alexa Anti-Mouse 488 lot no. 898230 and Alexa Anti-Rabbit 568 lot no. 757102, Invitrogen) diluted 1:1000. It should be noted that the TH antibody used only detects TH1 in the zebrafish (Chen et al., 2009).

### Microscopy and imaging

*In situ* hybridization samples were observed under a Leica DM IRB inverted microscope with an attached Leica DFC490 camera. Multifocus images were taken using Leica application suite version 2.7.0 software (Leica Microsystems CMS GmbH, Switzerland).

Immunofluorescent samples were visualized using a Leica SP2 AOBS confocal microscope (Leica Microsystems GmbH, Mannheim, Germany). The images were acquired using a HC PL APO 20×/0.70 CS objective. For detection of the fluorophores, a 488 nm argon laser and a 568 nm diode laser were used. The emission was collected at 500–550 nm for the 488 nm laser and at 600–700 nm for the 568 nm laser. The distance between stack planes was set at approximately 1 μm. The image stacks were imported to Fiji [open source imaging software (Schindelin et al., 2012)] for cell number quantification. Statistical analysis of cell numbers was done using One-Way ANOVA.

### Neurotransmitter measurement by high-performance liquid chromatography

For analysis of catecholamine levels 15–20 whole larvae were sonicated in 10 volumes of 2% perchloric acid, centrifuged for 30 min at 15,000 g after which 10 μL of filtered supernatant was injected into high-performance liquid chromatography (HPLC) system equipped with a Waters Concorde electrochemical detector set to a potential +0.80 V, column oven and a column Gemini C18 5 μm 150 × 4.60 mm (Phenomenex, Torrance, CA, USA). The mobile phase consisted of purified water with 8% methanol, 50 mM citric acid, 1.5 mM 1-octanesulfonic acid, 0.05 mM EDTA, and 50 mM phosphoric acid. The column temperature was set at 37°C and the flow rate at 1 ml/min. System control, data acquisition and analysis were performed using Waters Empower software (Waters, Milford, MA). Concentrations of the catecholamines and metabolites were calculated from standard curves which were linear from 10 nM to 1 μM. The protocol for histamine measurement has been described earlier (Eriksson et al., 1998). In order to normalize the data, sample protein concentration was measured using the Pierce^©^ BCA Protein Assay Kit (Thermo Fisher Scientific INC., Rockford, IL, USA). The measured concentration was normalized per protein, and the data is reported as percent of the average of the control group. Statistical analysis was done using One-Way ANOVA followed by Tukey's *post hoc* test.

## Results

### The effect of ethanol on swimming behavior

**Figure 1:**
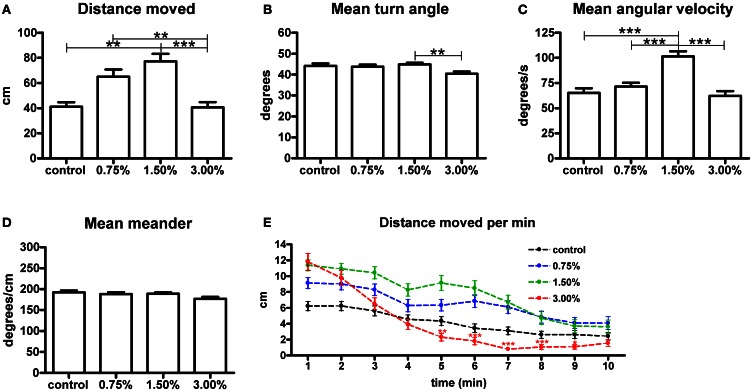
**The effect of 0.75%, 1.50% and 3.00% ethanol concentration on locomotion in larval zebrafish during the first 10 minutes after exposure.** A significant increase can be seen in the total distance moved **(A)** up to a 1.50% concentration, with a 3.00% concentration having no apparent change on total distance moved [*F*_(3, 213)_ = 8.304, *p* < 0.0001]. The angular velocity **(C)** is also significantly altered in the 1.50% group [*F*_(3, 213)_ = 16.18, *p* < 0.0001]. We could also see a significant difference in turn angle **(B)** between the 1.50% and 3.00% groups, but this observation was inconsistent across repeats and therefore unlikely to be of any relevance [*F*_(3, 213)_ = 4.046, *p* = 0.0080]. No differences are seen in meander **(D)** [*F*_(3, 213)_ = 2.331, *p* = 0.0753]. Statistics were done using 1-way ANOVA followed by Tukey's *post-hoc* test, ^*^p < 0.05, ^**^p < 0.01, ^***^p < 0.001. When plotting the data as total distance moved per minute **(E)**, the initial stimulatory effect of the 3.00% concentration can clearly be seen. Both time [*F*_(9, 1917)_ = 89.02, *p* < 0.0001] and treatment concentration [*F*_(3, 213)_ = 5.865, *p* = 0.0007] had extremely significant effects on the total distance moved. There was also significant interaction between the time and treatment concentration [*F*_(27, 1917)_ = 4.396, *p* < 0.0001], but this is mostly attributable to the strong biphasic effect observed for the 3.00% group. Statistics were done using 2-way repeated measures ANOVA followed by Bonferroni's *post-hoc* test. The numbers of larvae in each group were as follows: control: *N* = 47, 0.75%: *N* = 56, 1.50%: *N* = 61, 3.00%: *N* = 53.

We measured the total swimming distance and the mean values for meander, turn angle and angular velocity during the 10-min treatment period (Figure 1). The 1.50% ethanol concentration increased the total swimming distance significantly (*p* < 0.05). A 3.00% concentration did not have any effect on the total distance moved. We also observed that the group treated with 1.50% ethanol showed a significant increase in mean angular velocity (*p* < 0.001), while the other treatment concentrations did not have any effect on this parameter. Mean meander and turn angle remained unchanged across all groups, except for a significant yet small change in turn angle observed between the 1.50 and 3.00% groups that was inconsistently observed between trials. We therefore do not find it to be of any relevance (Figures 1B,D).

In order to further understand the effect on the total distance moved, we analysed the parameter in 1-min intervals (Figure 1E). This revealed that the 3.00% concentration actually had a strong stimulatory effect on locomotion during the first few minutes of the treatment, but this effect subsided quickly and locomotor activity clearly diminished in the group for the remaining duration of the trial [time effect, *F*_(9, 1791)_ = 75.20, *p* < 0.0001]. This sedative effect explained why there was no apparent effect on the total distance moved throughout the trial. This sedative effect was not observed for the 0.75 and 1.50% groups. The magnitude of the initial increase in locomotor activity appeared to be dose-dependent [treatment effect, *F*_(3, 1791)_ = 8.21, *p* < 0.0001].

### Changes in tyrosine hydroxylase 1, tyrosine hydroxylase 2, and histidine decarboxylase transcript levels

**Figure 2:**
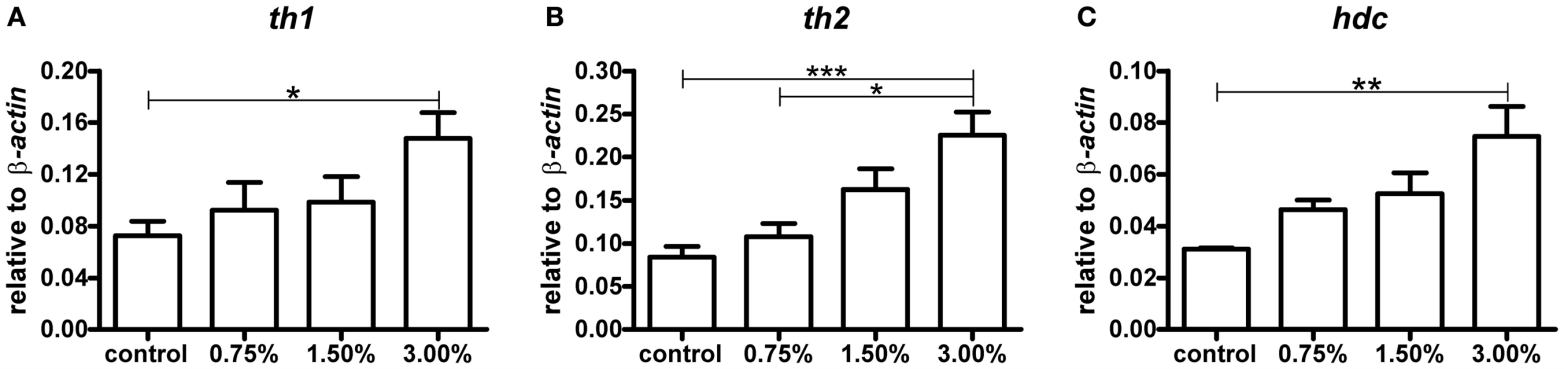
**The levels of *th1, th2*, and *hdc* mRNA after a 10-min ethanol treatment.** A clear dose-dependent trend can be seen for all mRNA species analysed. Statistical significance was analysed using One-Way ANOVA followed by Tukey's *post-hoc* test. **(A)** *F*_(3, 17)_ = 3.439, *P* = 0.0405; **(B)** *F*_(3, 17)_ = 8.489, *P* = 0.0011; **(C)** *F*_(3, 17)_ = 5.515, *P* = 0.0079, ^*^p < 0.05, ^**^p < 0.01, ^***^p < 0.001 according to the *post-hoc* test. *N* = 6 except for the 0.75% group, for which *N* = 3.

The amount of mRNA for each of the three rate-limiting enzymes in the biosynthesis of dopamine and histamine showed a clear dose-dependent increase trend following exposure to ethanol (Figure 2). The zebrafish has two tyrosine hydroxylase isoforms, TH1 and TH2 (Chen et al., 2009). Both *th1* and *th2* mRNA levels were significantly increased by the 3.00% ethanol treatment (*p* < 0.05 for *th1, p* < 0.01 for *th2*). The increase in *hdc* mRNA was also significant (p < 0.01). These results indicate that ethanol directly or indirectly activates synthesis of the enzymes regulating the synthesis of dopamine and histamine in zebrafish almost immediately following exposure.

### Immunohistochemistry and *in situ* hybridization

**Figure 3:**
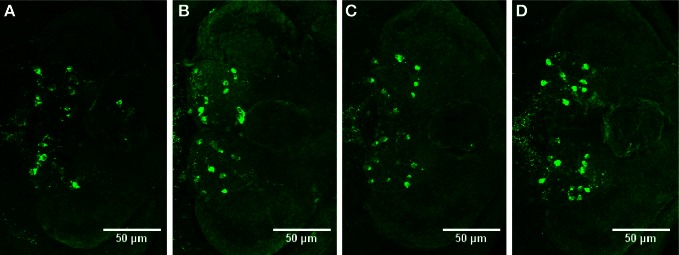
**Distribution of histamine-immunoreactive cells after a 10-min ethanol treatment. (A)** Control, **(B)** 0.75% ethanol, **(C)** 1.50% ethanol, **(D)** 3.00% ethanol. No changes are seen between the groups, which are further confirmed by cell counting statistics in Figure 4. *N* = 13–15 larvae per treatment group.

**Figure 4:**
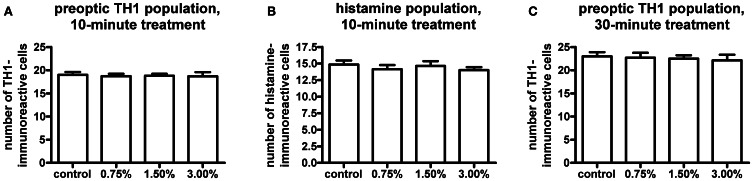
**Cell numbers in the histaminergic and preoptic dopaminergic populations after the ethanol treatment.** A 10-min ethanol treatment had no effect on either population. In order to see if any change was occurring after a longer treatment, the preoptic dopaminergic population was further analysed after a 30-min treatment. The longer treatment also did not show any changes in cell numbers. Statistical analysis was done using One-Way ANOVA. **(A)** *F*_(3, 52)_ = 0.05260, *p* = 0.9839; **(B)** *F*_(3, 52)_ = 0.4550, *p* = 0.7149; **(C)** *F*_(3, 33)_ = 0.1273, *p* = 0.9432. *N* = 13–15 for the 10-min treatment, *N* = 8–10 for the 30-min treatment.

We observed no change in the expression pattern of *th1, th2*, and *hdc* by *in situ* hybridization in any of the groups after a 10-min treatment (data not shown). These results were supported by immunohistochemistry, which also showed no changes in distribution patterns of TH1- and histamine-immunoreactive cells and fibers (Figure 3, data not shown for TH1). This was verified by counting the number of histamine- and TH-immunoreactive cells in the hypothalamus and preoptic group [groups 3 and 4 as defined by Sallinen et al. (2009b)], respectively. No change in cell numbers was seen (*p* > 0.05, Figure 4).

**Figure 5:**
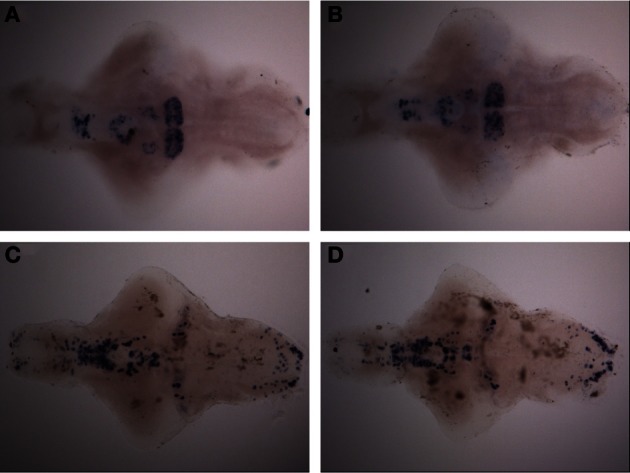
**Expression pattern of *th1* and *th2* after a 30-min ethanol treatment. (A)** *th2* expression pattern after 30-min of 1.50% ethanol. **(B)** *th2* expression in the control group. **(C)** *th1* expression pattern after 30-min of 1.50% ethanol. **(D)** *th1* expression in the control group. No differences can be seen between the groups. *N* = 10 larvae per treatment group.

**Figure 6:**
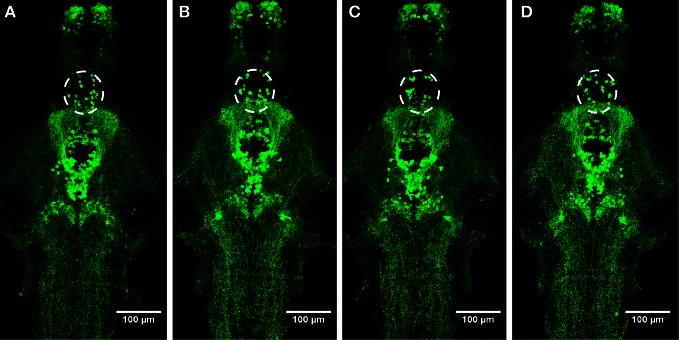
**Distribution of TH1-immunoreactive cells and fibers after a 30-min ethanol treatment. (A)** Control, **(B)** 0.75% ethanol, **(C)** 1.50% ethanol, **(D)** 3.00% ethanol. No changes are seen between the groups. *N* = 8–10 larvae per treatment group.

In order to determine if a prolonged treatment would alter the morphology of the histaminergic and dopaminergic systems, we repeated the *in situ* hybridization using a 30-min ethanol treatment. For this experiment, only the 1.50% ethanol dose was used, based on the acquired behavioral data. The longer treatment period did not show any difference in the expression patterns of *hdc, th1*, and *th2* between the 1.50% group and the control (Figure 5, data not shown for *hdc*). Immunohistochemistry also showed that the number and distribution of TH1immunoreactive cells remained unchanged in the preoptic group after the extended ethanol treatment (*p* > 0.05, Figures 4, 6).

### Changes in dopamine and histamine levels observed by HPLC

**Figure 7:**
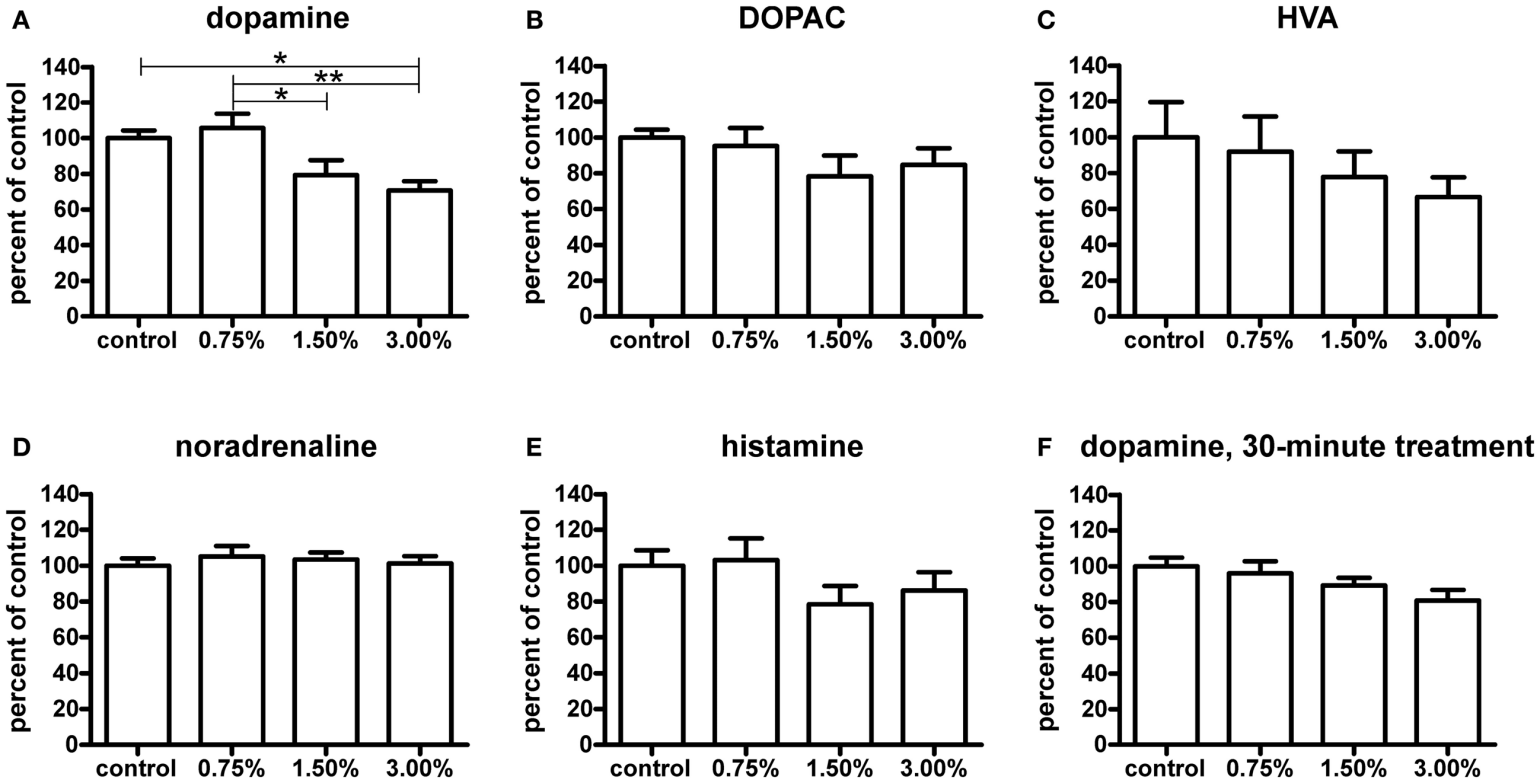
**Neurotransmitter levels relative to control group measured by HPLC after a 10-minute ethanol treatment.** The dopamine **(A)** levels were significantly decreased with a 1.50% and 3.00% treatment, whereas a 0.75% does not have any visible effect [*F*_(3, 24)_ = 6.174, *p* = 0.0029]. The levels of HVA **(C)** show a slight non-significant decreasing trend across treatment groups [*F*_(3, 22)_ = 0.7453, *p* = 0.5366], while DOPAC **(B)** levels are unchanged [*F*_(3, 24)_ = 1.140, *p* = 0.3531]. Noradrenaline **(D)** and histamine **(E)** levels were not significantly affected [noradrenaline: *F*_(3, 24)_ = 0.2692, *p* = 0.8470; histamine: *F*_(3, 24)_ = 1.246, *p* = 0.3149], but a slight decrease trend of the histamine levels can be seen in the 1.50% and 3.00% groups. After a 30-minute treatment **(F)**, the observed decrease in dopamine levels was smaller and not significant [*F*_(3, 25)_ = 2.417, *p* = 0.0901]. Statistical analysis was done using 1-way ANOVA followed by Tukey's *post-hoc* test. ^*^p < 0.05, ^**^p < 0.01, according to the *post-hoc* test. *N* = 7, except for the control group and 3.00% group in the HVA data where *N* = 6.

**Figure 8:**
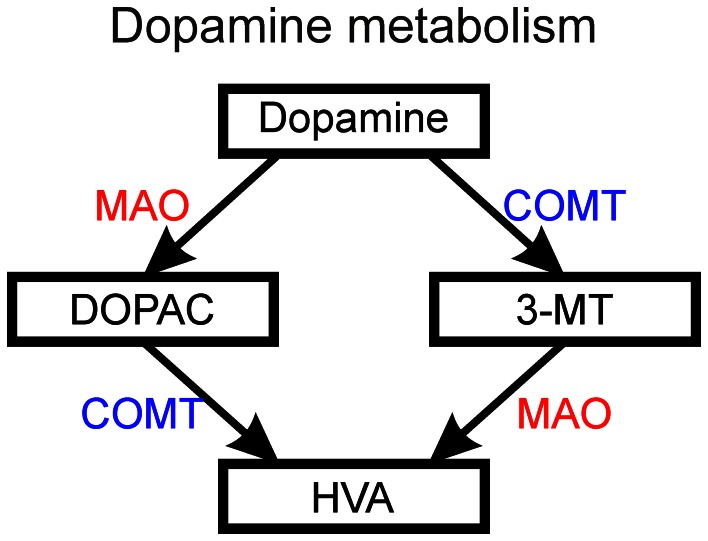
**The metabolism of dopamine into DOPAC (3,4-dihydroxyphenylacetic acid) and 3-MT (3-methoxytyramine) followed by metabolism of these intermediate products into HVA (homovanillic acid) by the action of MAO (monoamine oxidase) and COMT (catechol-O-methyltransferase)**.

The higher ethanol concentrations resulted in significantly decreased dopamine levels after a 10-min treatment (*p* < 0.05, Figure 7A). Curiously, the 0.75% group did not show a decrease in dopamine. None of the dopamine metabolites analysed [3,4-dihydroxyphenylacetic acid (DOPAC) and homovanillic acid (HVA)] showed any significant changes (Figures 7B,C). For the metabolic pathways of dopamine, please refer to Figure 8. HVA levels showed a slight dose-dependent decreasing trend, but this observation did not reach statistical significance (*p* > 0.05). The levels of other metabolites remained consistent across all groups. In order to see if the upregulation of *th1* and *th2* observed after a 10-min treatment would counteract the decrease in dopamine observed, we measured the dopamine levels again after a 30-min treatment (Figure 7F). At this point, the difference in dopamine levels observed between the groups was smaller and statistically not significant, indicating that the dopamine loss might indeed be compensated.

Out of other neurotransmitters, histamine, and noradrenaline were measured. Noradrenaline levels were unaffected by the treatment (Figure 7D). Only a non-significant decreasing trend in histamine levels (*p* > 0.05) was seen (Figure 7E).

## Discussion

Our results showed that ethanol concentrations of 0.75 and 1.50% have an acute stimulatory effect on locomotion in larval zebrafish, while a 3.00% concentration resulted in a brief increase in activity, quickly followed by a strong decrease of locomotion. This corresponds well with results obtained from earlier studies (Lockwood et al., 2004; Macphail et al., 2009; De Esch et al., 2012), and shows that the fish strain used here displays a concentration-dependent increase in locomotor activity following exposure to ethanol. Out of these earlier studies, only Lockwood et al. (2004) have analysed the movement activity immediately after administration of ethanol, showing a large difference in the magnitude of the ethanol-induced stimulation between the AB and WIK wild-type strains of zebrafish (Lockwood et al., 2004). Our Turku strain reacted similar to the WIK strain, showing a rather modest increase in locomotor activity. The initial stimulatory effect we observed for the 3.00% treatment could be explained by a similar biphasic effect that was observed in a study with mice (Crabbe et al., 1982), where ethanol doses cause a similar effect for C57BL/6N mice. Another possibility is that the initial response to ethanol would be caused by the presence of an irritating substance. This was, however, also investigated by Lockwood et al. (2004), who showed that a 1.50% methanol treatment had no effect on locomotion (Lockwood et al., 2004). We therefore find it reasonable to interpret the observed stimulation as an effect attributable to the pharmacological properties of ethanol and not as an unspecific reaction to a noxious substance. Our data also suggests that ethanol changes the swimming pattern of larval zebrafish, as shown by the increase in mean angular velocity for the 1.50% group. This might indicate an impairment of motor coordination after a 1.50% ethanol treatment, which is a well-known effect of ethanol (Carta et al., 2004).

The main focus of our study was to characterize the dynamic changes in in key neurotransmitters associated with locomotor activation following ethanol exposure. The stimulatory effect of ethanol is known to be linked to the activation of mesolimbic dopaminergic pathways in mice (Meyer et al., 2009). TH is the rate-limiting enzyme of dopamine synthesis. Zebrafish have two isoforms of TH called TH1 and TH2. The distribution of *th1* and *th2* cells in the zebrafish brain show a complementary pattern, with *th1* being the dominant isoform in the brain (Chen et al., 2009). Since an increase in *th2* has been shown to be affected by different states of behavior (Pavlidis et al., 2011), we hypothesized that ethanol might affect either enzyme only. This was, however, not the case, as we saw an increase in transcript numbers of both *th* genes. It would therefore seem that ethanol has a general stimulatory effect on dopamine synthesis in the zebrafish, since these TH forms are expressed in different neuron populations with different projections (Chen et al., 2009). This is also supported by our data obtained by *in situ* hybridization, which showed that the patterns of *th1* and *th2* expression in the brain remained unchanged, and immunohistochemistry, which showed that the number and pattern of TH1-immunoreactive cells remained unaffected.

As different populations of dopaminergic neurons project widely to key areas involved in motor regulation, for instance the subpallium (Rink and Wullimann, 2001; Tay et al., 2011), the nucleus of the medial longitudinal fascicle (McLean and Fetcho, 2004) and even the spinal motor neurons themselves (McLean and Fetcho, 2004), it is of key interest to know if the stimulatory effect of ethanol is mediated by one or several of these systems, or through a diffuse activation of the dopaminergic network. In mammalian models, the activation of locomotion has been strongly linked to effects on the VTA dopaminergic neurons, demonstrating, for instance, increased activation of VTA neurons (Gessa et al., 1985) increased *th* mRNA levels in the VTA (Oliva et al., 2008) and increased dopamine release in VTA target areas (Di Chiara and Imperato, 1986). We were unable to measure the release of dopamine in different brain areas due to the small size of the zebrafish brain, but our data obtained by qPCR, immunohistochemistry and *in situ* hybridization supports the theory of a diffuse activation of the dopaminergic network, as discussed in the previous section. It should, however, be noted that our analysis on the role of TH2 in this process is incomplete, as we did not have access to a working characterized TH2 antibody during this study. Also, although the activation of the dopaminergic system might be general, this does not directly imply that all possible pathways participating in the stimulation of locomotion are activated. Our results give reason for future studies further investigating this effect. *In situ* hybridization and immunocytochemistry showed that there was no change in cell numbers expressing the *th* transcripts following ethanol. The activation thus occurred in cells which also under control conditions express *th1* or *th2*.

The higher ethanol concentrations seemed to have a decreasing effect on dopamine levels in the zebrafish after a 10-min treatment. The increase in *th1* and *th2* transcription might be a part of a mechanism to increase synthesis of TH and therefore the synthesis of dopamine, which is supported by our data showing no significant differences between treatment groups after a 30-min treatment. A decrease in dopamine also implies that ethanol is stimulating dopamine release, which is a well-known phenomenon in rodents (Di Chiara and Imperato, 1986, 1988). The increase in dopamine release would explain the stimulating effect on locomotion. We were, however, unable to directly demonstrate an increase in dopamine release, as microdialysis is not feasible in larval zebrafish. Although earlier studies with zebrafish have demonstrated an increase in dopamine following an acute ethanol dose (Chatterjee and Gerlai, 2009; Gerlai et al., 2009), these results were obtained using adult zebrafish and lower ethanol treatment doses. Since the pharmacokinetic differences between larval and adult zebrafish have yet to be elucidated, it is difficult to compare our results and results obtained in adult fish. In our study, only the 1.50 and 3.00% ethanol concentrations resulted in a decrease in dopamine levels, while a 0.75% concentration had no significant effect. It is therefore entirely possible that only higher ethanol doses cause dopamine levels to actually decrease, while the increased synthesis implicated by increased *th1* and *th2* transcription compensates the decrease at lower doses. Curiously, we did not see an increase of any of the dopamine metabolites, which would be expected if dopamine levels were decreased due to increased release and metabolism. Instead, we saw that HVA levels actually decreased slightly in a dose-dependent manner. The kinetics of the metabolism of catecholamines in zebrafish is not known, and more research will be necessary in order to explain this change. One might argue that the decrease in dopamine would indicate some kind of acute toxic effect on dopaminergic neurons. We find this to be unlikely, however, as we could not see any change in the expression pattern of *th1* and *th2* or TH1-immunoreactive neurons even after a longer, 30-min ethanol treatment.

Although ethanol treatments of similar concentration have proven to be teratogenic in younger zebrafish (Reimers et al., 2004; Arenzana et al., 2006), there is evidence of changes in the expression of alcohol dehydrogenase 3 (ADH3) during fish development, which implicates possible age-dependent differences in the metabolism and toxicity of ethanol on zebrafish (Dasmahapatra et al., 2001). Internal ethanol concentrations in the 0.75–3.00% range are likely to be lethal in mammals. It was shown by Lockwood et al. that a 20-min treatment of 7-day-old larval zebrafish resulted in internal ethanol concentrations of 25 mM (0.12%) and 71 mM (0.33%) after treatment with 1.5 and 3.0% ethanol solutions, respectively (Lockwood et al., 2004). Internal concentrations around this range have been reported in studies with mice (Nuutinen et al., 2011), and the use of similar ethanol concentrations in other studies with zebrafish (Lockwood et al., 2004; Macphail et al., 2009) further justifies the use of high ethanol concentrations in the treatment solutions.

A novel discovery in our study was that ethanol increased the transcription of HDC, the rate-limiting enzyme of histamine synthesis. Recent studies in mice have demonstrated that histamine indeed is essential in ethanol-induced locomotor activation and also in the associated reward-mechanism (Nuutinen et al., 2010, 2011). Again, the morphology and number of the histaminergic system was unaffected, as verified by *in situ* hybridization and immunohistochemistry, which would support the assumption that the increase is due to an upregulation of the *hdc* gene in cells which express hdc under normal conditions. In zebrafish, the histaminergic neurons show life-long plasticity and there numbers are regulated by presenilin 1 through Notch1 (Sundvik et al., 2013). They are essential in regulation of e.g., dark flash swimming response (Sundvik et al., 2011) and histamine regulates vigilance or anxiety-like behaviors (Peitsaro et al., 2003). Also in rodents activity of histaminergic neurons is linked to increased vigilance and cognitive capacity (Haas and Panula, 2003; Anaclet et al., 2009). Further studies are necessary in order to elucidate the purpose and effects of the increase in *hdc*.

In conclusion, this study shows evidence that an acute ethanol treatment increases locomotor activity with moderate doses of ethanol in larval zebrafish. At high concentration, an acute increase in locomotor activity is associated with a decline in dopamine levels and induction of *th1, th2*, and *hdc* without any change in the anatomy of the dopaminergic and histaminergic systems, indicating that ethanol might have a diffuse stimulatory effect on these systems in zebrafish. In addition, this study provides evidence that a 1.50% ethanol concentration causes impairment of motor control in larval zebrafish, as seen by the increase in fish angular velocity. The decline in dopamine levels after a 10-min treatment might result from strongly increased dopamine release followed by upregulation of both *th* forms, resulting in partially normalized dopamine levels after a 30-min treatment. This provides a basis for further study, in order to fully understand the effect of ethanol on the dopaminergic and histaminergic systems in zebrafish.

### Conflict of interest statement

The authors declare that the research was conducted in the absence of any commercial or financial relationships that could be construed as a potential conflict of interest.
